# Blockchain-Based Incentive Mechanism for Electronic Medical Record Sharing Platform: An Evolutionary Game Approach

**DOI:** 10.3390/s25061904

**Published:** 2025-03-19

**Authors:** Dexin Zhu, Yuanbo Li, Zhiqiang Zhou, Zilong Zhao, Lingze Kong, Jianan Wu, Jian Zhao, Jun Zheng

**Affiliations:** 1 School of Cyberspace Science and Technology, Beijing Institute of Technology, Beijing 100081, China; 2 College of Computer Science and Technology, Changchun University, Changchun 130022, China

**Keywords:** blockchain, evolutionary game theory, incentive mechanism, electronic medical records

## Abstract

As the medical information systems continue to develop, the sharing of electronic medical records (EMRs) is becoming a vital tool for improving the quality and efficiency of medical services. However, during the process of sharing EMRs, establishing mutual-trust relationships and increasing users’ participation are urgent problems to be solved. Current solutions mainly focus on incentive mechanisms for users’ honest and active participation, but often ignore the potential impact of research institutions’ behavior on users’ trust and participation. To address this, this paper proposes an incentive mechanism based on evolutionary game theory. It combines the unchangeable nature of blockchain and the dynamic adjustment characteristics of evolutionary games to build a secure and trustworthy incentive system. This system considers the potential malicious behaviors of both users and research institutions, encouraging research institutions to protect users’ privacy, reduce users’ concerns, and guide users to actively contribute data. At the same time, it ensures data security and system trust through clear rewards and punishments. Based on this, we have carried out a comprehensive simulation using game theory. The results confirm that our designed incentive mechanism can effectively achieve its expected goals.

## 1. Introduction

With the advent of the big data era, medical data sharing has become an inevitable trend in the healthcare industry. By breaking down information silos among medical institutions and integrating resources and knowledge from diverse sources, this practice has significantly enhanced the quality and efficiency of healthcare services [1,2]. As the core component of medical data, the sharing of Electronic Health Records (EHRs) carries profound significance. EHR sharing facilitates the optimized allocation of medical resources, promotes collaborative efforts among healthcare institutions, and enables highly efficient resource utilization [3]. However, shared patient EHRs are frequently compromised during sharing, transmission, or utilization due to improper management or malicious attacks, resulting in information leakage and data tampering [4].

Blockchain technology, leveraging its distributed ledger, cryptographic algorithms, and immutability characteristics, now provides an effective solution to this challenge [5]. Integrating blockchain into EHR sharing systems enables the creation of secure sharing environments that ensure tamper-proof transmission and storage of medical records. This integration significantly enhances the security and trustworthiness of data sharing, establishing a robust foundation for secure medical data circulation [6]. Researchers have progressively advanced blockchain applications in EHR sharing through in-depth investigations, particularly in strengthening transmission privacy [7,8] and ensuring secure storage mechanisms [9,10]. These studies collectively demonstrate the substantial potential of blockchain technology in advancing EHR sharing systems [11].

While blockchain technology provides prominent advantages for EHR sharing—such as tamper resistance and enhanced privacy protection—collaboration and incentive mechanisms among distributed entities remain latent risks. The stable and efficient operation of blockchain-based EHR sharing systems fundamentally relies on active collaboration and deep engagement from these distributed participants. Nevertheless, in practical implementations, dynamically evolving environments and unpredictable entity behaviors pose significant challenges to system performance [12]. For instance, research institutions may maliciously disclose patient data after receiving EHRs, causing user losses that ultimately undermine their willingness to actively engage in data sharing. Conversely, users fearing privacy breaches by these institutions might either submit falsified data or abstain from participation entirely in EHR sharing systems.

Hence, establishing effective incentive mechanisms constitutes the cornerstone for ensuring service equity and systemic efficiency. By deploying a hybrid reward–penalty strategy, such mechanisms incentivize all participants to deliver high-quality services, substantially mitigate risks of data breaches and tampering, and prevent both malicious competition and inefficient resource utilization [13,14].

Therefore, establishing an incentive system that can both satisfy the interests of all parties and enhance system efficiency is of great importance for promoting data sharing, ensuring service quality, maintaining system security, and facilitating the long-term stable development of the wise medical system. The limitations of current research lie in the static nature of incentive mechanism design and insufficient scenario-specificity.

Existing incentive mechanisms face three-fold fitness challenges. Firstly, current mechanisms mainly focus on stimulating data contributors to provide authentic and reliable data [15,16], but fail to build an effective behavioral constraint framework for data users, resulting in a structural imbalance of one-way incentives. Secondly, the rapidly increasing frequency, scale, and economic losses of medical data leaks [17] reveal the weakness of current mechanisms in preventing such leaks. Thirdly, in dynamic game-theory scenarios, although some studies have tried to optimize the situation through token-based rewards and punishments [18,19] or dynamic reputation mechanisms [20,21], these solutions have not adequately considered the strategic evolution characteristics of participating roles in medical scenarios.

To address the above challenges, this study introduces evolutionary game theory as a theoretical framework. By simulating the long-term strategy evolution of groups, it can effectively depict the strategy adjustment process of multiple participants in medical data sharing. Compared with traditional game theory, its dynamic replicator equations are especially suitable for analyzing the gradual behavioral evolution of participants in a blockchain environment [22]. In a blockchain-supported electronic medical record sharing system, evolutionary game theory can be applied to analyze strategic interactions among participants, enhance data security, promote cooperation, and design more effective incentive mechanisms [23].

The main contributions of this paper can be summarized as follows:

(1) Incentive Mechanisms for Users and Research Institutions in the EMR Platform: Combining the tamper-proof feature of blockchain and the dynamic adjustment feature of evolutionary game, we construct a secure and trustworthy incentive system. This system takes into account the potential malicious behaviors of both users and research institutions, breaking through the previous limitation of only focusing on users’ malicious behaviors. By incentivizing research institutions to protect the privacy of users’ medical records, we can alleviate users’ concerns and encourage them to contribute data actively. At the same time, rewards are given to those who abide by the rules, and punishments are imposed on violators, ensuring data security and system trust.

(2) Game-theoretic Analysis of the Incentive Mechanism in the EMR Platform: By building a behavior analysis platform based on blockchain, we provide data support for evolutionary games. We use evolutionary game theory to analyze the incentive mechanism, accurately grasp the interaction relationship between users and research institutions, and dynamically optimize the incentive parameters according to the evolutionary laws, improving the effectiveness of the incentive mechanism.

(3) Simulation of the Incentive Mechanism in the EMR Platform: We break the single-data-contribution incentive model and design a multi-party collaborative incentive model. Differentiated incentives are given according to the compliance of users’ data usage and the efficiency of research institutions’ achievement transformation, fully mobilizing the enthusiasm of all parties and creating a fair and stable system environment. We verify the results through game-theoretic simulation, visualize the system evolution process, and enhance the reliability of the research results.

## 2. Related Work

### 2.1. EMRs Using Blockchain Technology

In recent years, blockchain technology has emerged, and its application in EMRs platforms has become a research hotspot [24]. The encryption and decentralized storage features of blockchain powerfully safeguard data security and privacy. For example, the new Ethereum-based system proposed in reference [25] gives users secure control over their medical data. It addresses the key challenges in the implementation of healthcare blockchain, effectively avoids the risks of data loss and leakage, and fully meets the strict requirements for medical data privacy protection.

In terms of data sharing and interoperability, blockchain has broken through the barriers of traditional medical systems. The EMRs platform based on consortium blockchain enables medical institutions to maintain medical record data through the consensus mechanism, achieving the real-time sharing of medical records and enhancing the efficiency and quality of medical services. For instance, when a patient is transferred, the receiving hospital can quickly obtain the complete medical record, thus avoiding repeated examinations. The non-tamperable feature of blockchain ensures the authenticity and traceability of medical data. Every data operation is recorded in the blockchain, forming a non-tamperable time chain, which provides a reliable basis for handling medical disputes and tracing scientific research data.

However, blockchain also faces some challenges in its application in EMRs platforms. Among them, the imperfect incentive mechanism restricts its development to a certain extent. At present, quite a number of studies have been dedicated to exploring incentive measures. For example, Gan et al. [26] employed users as supervisors and motivated users to actively participate in data sharing by granting them the authority to manage medical data. Another example is that Shen et al. [18] adopted a Shapley-value-based scheme as the incentive mechanism of the system. Aiming at the fair distribution of the benefits from shared data, this mechanism encourages both users and miners to participate in data sharing. Liu et al. [19] employed a multi-dimensional and multi-faceted reputation evaluation mechanism, along with a token-based reward and punishment mechanism for research institutions, to encourage active participation from both users and medical institutions. Purohit et al. [20] proposed establishing a reputation scoring system by considering the historical feedback and risk assessment of medical data users and providers. This aims to encourage cooperation among organizations and build a trust-based collaboration mechanism. Zou et al. [21] designed special key blocks and micro-blocks for users to store their Electronic Medical Records, aiming to encourage users to participate. On this basis, a corresponding reputation system was constructed to encourage the participation of medical institutions. However, when dealing with the complex and diverse behaviors of participants, these incentive mechanisms still show certain limitations.

Although these studies have proposed a variety of incentive mechanisms, the comparative analysis presented in Table 1 reveals that existing schemes still exhibit significant limitations in terms of incentive differentiation and dynamic adaptability.

### 2.2. Evolutionary Game Theory

Evolutionary game theory is an important branch of game theory, integrating theories from evolutionary biology and rational-choice economics. It focuses on analyzing the evolutionary process of strategies in a competitive environment. Within this theoretical framework, a core concept is the evolutionarily stable strategy (ESS). It describes a strategic state where, once the majority of members in a population adopt this strategy, it can effectively resist the invasion of any alternative strategies and maintain its stability in the long run. This means that when a strategy becomes an ESS, the individuals in the population gain an adaptive advantage from adopting this strategy, making it unlikely for them to switch to other strategies, thus ensuring the continuous existence of this strategy within the population [27].

Nowadays, many scholars also use evolutionary game theory to study different aspects of blockchain system. For example, Liu et al. [28] utilized evolutionary game theory to analyze the process of mining pool selection in the blockchain system network, revealing the conditions under which the network permits a unique evolutionarily stable state. Sun et al. [29] employed evolutionary game theory to construct an incentive mechanism in a readable blockchain system and conducted a comprehensive analysis based on game theory. Zhang and Wu [30] utilized evolutionary game theory to examine the cooperation mechanism among participants in the blockchain system network, emphasizing the need to reduce the profit expectations of malicious participants to maintain the integrity of the ledger. Xuan et al. [31] used evolutionary game to conduct an equilibrium analysis of data sharing in the big-data era, explored and analyzed the evolutionary game process of data sharing, and designed a smart contract to implement the incentive mechanism. However, the models they considered were not specific enough. Therefore, improvements to the models and analyses are needed in different scenarios. Jie et al. [32] proposed a different solution strategy for the possible prisoner’s dilemma in the field of blockchain system data sharing by applying evolutionary game theory. They not only conducted an analysis in the benchmark case of discrete strategies in two-player games, but also extended it to the context of continuous strategies in multi-player games. In this process, they paid particular attention to the behaviors of all individual data providers and designed an optimal zero-determinant (ZD) strategy. These strategies can effectively guide individuals on how to make decisions that best serve their own interests in different situations. At the same time, they also provide theoretical support for the stable operation of the entire system.

However, current research on deeply integrating evolutionary game theory into the incentive mechanism for EMRs platform is still in its infancy. There is a need to deeply analyze the decision-making behaviors of different roles, and further improve the incentive mechanism based on the results of these analyses. This is not only of great significance for enhancing the security of the EMRs platform and ensuring its long-term stable operation, but also will strongly promote the accelerated development of the medical digitization process.

## 3. Preliminaries

### 3.1. Evolutionary Game Theory

Since the 1990s, with the times evolving, to compensate for the shortcomings of traditional game theory, evolutionary game theory emerged and has been studied and valued by many scholars. It can describe the dynamic game process among game entities and analyze the evolutionarily stable strategies of specific dynamic processes using game theory. Methodologically, differing from traditional game theory, it focuses on dynamic equilibrium strategies rather than static ones. In 1970, the concept of evolutionary stable strategy was first proposed by Smith and Price [33]. In the real world, inspired by biological evolution, scholars developed evolutionary game theory based on traditional game theory. It can depict and explain the gradual evolution in biological processes. In recent years, as its research scope expands and content becomes more complex, many experts have applied it to computer science, finance, and biology. With solid theoretical foundations, evolutionary game theory involves analyzing specific problems, identifying key factors, selecting scientific indicators, and building models to describe the dynamic learning of different rational groups. By analyzing equilibrium points, it assesses local stability, explores evolutionarily stable strategies, and determines if dynamic models converge to Nash equilibrium [34].

In analyzing the interest decisions of information-sharing entities, evolutionary game theory effectively depicts the relationships among game entities. Despite real-world uncertainties, the game models established through this theory can dynamically adjust via influencing factors. They optimize the control of entities affecting information-sharing strategies and explore interactive relationships and phenomena among game entities at the micro-level. By simulating game states through modeling amplification, it aims to achieve incentives and offers useful references for relevant countermeasures and suggestions.

### 3.2. Smart Contract

A smart contract is a computer protocol designed to informatize the dissemination, verification, and performance of contracts. In essence, it is self-executing contract terms deployed on a blockchain as code, automatically triggering actions when preset conditions are met, without intermediaries.

The concept was first introduced by Nick Szabo in 1994, centering on translating contract terms into executable code for automated performance and oversight. After blockchain emerged, smart contracts gained more attention and use. Blockchain’s decentralization, immutability, and transparency provide a solid foundation for their execution, ensuring that once deployed, the execution process and results follow predetermined rules, cannot be maliciously tampered with, and protect the rights and interests of transacting parties.

### 3.3. Reputation Mechanism

As a system for reducing transaction costs and optimizing resource allocation, the credit mechanism’s theoretical roots lie in the New Institutional Economics school. North’s theory of institutional change highlights the key role of informal institutions, with reputation-based constraints, a core element of social capital, cutting information-seeking and contract-enforcement costs during transactions [35]. Information economics further develops this idea. Akerlof’s “lemon market” model shows that in markets with information asymmetry, reputation mechanisms, by creating quality-signaling channels, can effectively resolve the adverse selection dilemma [36]. When market players establish a stable reputation-identification system, the market reaches “separating equilibrium”. High-quality suppliers gain market returns through reputation premiums, while low-quality ones are driven out by price mechanisms, establishing a positive-incentive cycle of “high-quality at high prices”.

## 4. System Model

To better analyze the impact of different behaviors of participating roles on the system in the sharing of Electronic Medical Records, we construct a blockchain-based electronic medical record sharing model for analysis. Our model encompasses the following entities: research institutions, users, the EMRs platform, and the blockchain system.

(1) Research institutions: They need users to share Electronic Medical Records to meet specific research objectives and offer remuneration to users.

(2) Users: They obtain economic rewards by sharing their Electronic Medical Records data.

(3) EMRs platform: It is responsible for task issuance and the review of Electronic Medical Records uploaded by users, as well as overseeing research institutions.

(4) Blockchain system: It stores the addresses of users’ Electronic Medical Records, records transactions, and implements the incentive mechanism through smart contracts.

**Figure 1:**
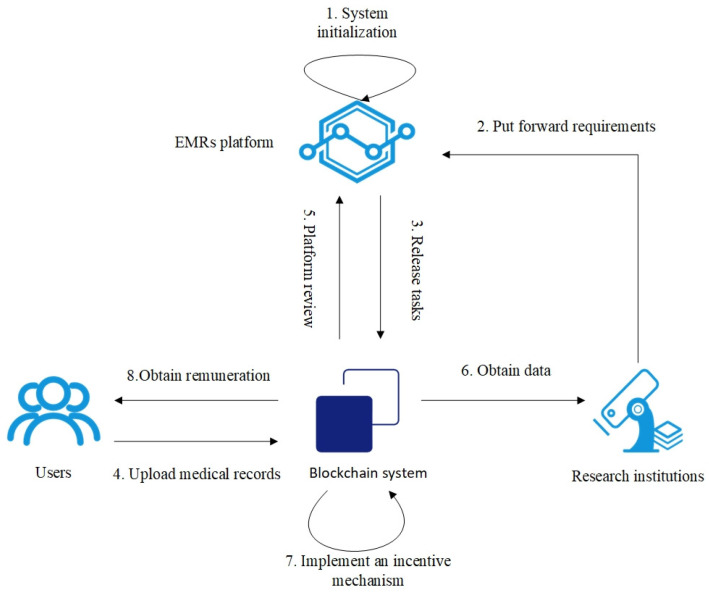
The systematic process of the Electronic Medical Record sharing platform.

The main processes of the system are shown in Figure 1 and include the following steps.

(1) System Initialization: The system sets initial reputation scores for research institutions and users.

(2) Submission of Requirements: Research institutions submit the task details of the required Electronic Medical Records to the EMRs platform.

(3) Task Publication: Based on the requirements, the EMRs platform publishes tasks in the blockchain system.

(4) User Upload of Medical Records: Eligible users upload the information to the IPFS (InterPlanetary File System), and then upload the hash address of the file and the medical record summary.

(5) Platform Review: The platform reviews whether the submitted records meet the task requirements. If they do, the research institution will be informed.

(6) Data Acquisition: The platform pays the remuneration and obtains the corresponding hash value from the blockchain system, thereby obtaining the Electronic Medical Records.

(7) Implementation of Incentive Mechanism: Through smart contracts, the incentive mechanism is implemented, and rewards or punishments are given according to the behaviors of all participants.

(8) Remuneration Acquisition: Users receive the remuneration for sharing their Electronic Medical Records.

In the system, we assume that the blockchain adopts a Reputation-based Practical Byzantine Fault Tolerance (RPBFT) consensus mechanism [19]. The RPBFT dynamically adjusts consensus committee members through periodic node reputation rankings. Nodes that engage in malicious behavior causing reputation degradation will be promptly deprived of their consensus participation privileges. This dynamic mechanism ensures the rapid integration of reputation evaluation results into the consensus process, preventing prolonged system obstruction by low-reputation nodes.

To maintain system integrity and operational efficiency, the platform establishes a participant reputation evaluation system. For user clients, the incentive strategy follows a linear correlation model: Reputation scores are positively correlated with compensation for Electronic Medical Record uploads. High-reputation users receive higher base reward coefficients, while low-reputation users face progressively diminishing returns. For research institutions, a priority scheduling mechanism is implemented—entities with superior reputation ratings receive prioritized processing of data requests in task queues, thereby accelerating data acquisition.

New participants obtain initial reputation values after identity verification. The user supervision module employs blockchain-based hash deduplication technology to intercept duplicate medical record submissions in real-time, triggering automatic score deductions while dynamically calibrating reputations through quality assessment feedback from data consumers. Research institution supervision focuses on confidentiality period compliance, where smart contracts automatically monitor data circulation records. Any on-chain resale activities detected during the confidentiality period (calculated from transaction completion) immediately incur reputation penalties, whereas compliance automatically grants reputation rewards upon period expiration.

We hereby define honest and malicious behaviors: For users, duplicate submissions of identical medical records constitute malicious behavior, whereas compliant uploads of non-repetitive records represent honest conduct. For research institutions, on-chain resale during the confidentiality period is strictly prohibited. Violations are deemed malicious, while protocol compliance maintains honest status.

## 5. Incentives for EMRs Systems

The corresponding descriptions of the parameters in this section are shown in Table 2.

### 5.1. Evolutionary Game Models

The model is defined as G(U,M,N), consisting of three components where *U* denotes the player, *M* represents the strategies available to the player, and *N* indicates the corresponding payoff for each strategy.

There are two players: the research organization and the user. These parties exhibit distinct differences in information acquisition and risk perception, demonstrating characteristics of limited rationality. Therefore, both parties base their strategic choices on the psychological expectations of perceived profit and loss values rather than the strategy’s direct benefits. Players have two strategy options each. The user’s strategies are honest compliance with rules (uploading genuine EMRs) or malicious uploads (submitting false EMRs), denoted as $M_{\mathrm{user}}\left(\mathrm{honest},\mathrm{malicious}\right)$. Similarly, research organizations choose between honest storage of EMRs or malicious leakage, denoted as $M_{\mathrm{research}}\left(\mathrm{honest},\mathrm{malicious}\right)$.

When the platform rewards a user for uploading EMRs, the compensation amount is adjusted according to the user’s current reputation score *T*. This adjustment aims to incentivize honest uploading by reducing rewards for users with lower reputation scores. To achieve this, we introduce a single-parameter smoothing function V(T) to modulate reward amounts. The function ensures $T_{\mathrm{starting}}$ serves as a baseline: When $T>T_{\mathrm{starting}}$, the reward increases proportionally; conversely, lower reputation scores result in reduced compensation. The constraint 0≤V(T)≤1 guarantees that rewards never exceed the research institution’s predefined maximum payout *R*.

The smoothing function is defined below in Equation (1):

(1) $$V\left(T\right)=\frac{1}{\left(1+e^{-K\left(T-T_{starting}\right)}\right)}$$

Users face two distinct costs when uploading EMRs. When uploading authentic medical records honestly, users incur time and privacy protection costs collectively termed the honest upload cost $C_{h}$. When submitting false/incomplete records maliciously, users primarily bear compilation costs termed the false upload cost $C_{p}$, with $C_{h}>C_{p}$ reflecting typical cost relationships.

User earnings combine direct platform rewards and reputation-based incentives. Research institutions gain benefits through (1) academic value $E_{h}$ from legitimate EMRs, (2) leakage gains $E_{l}$ from data disclosure, and (3) long-term reputational capital affecting sustainable resource access.

The EMRs platform implements dynamic reputation incentives with capped values: user reputation reward: $T_{\mathrm{uh}}\cdot M_{h}\cdot x$ (maximum $T_{\mathrm{uh}}\cdot M_{h}$); user reputation penalty: $T_{\mathrm{up}}\cdot M_{p}\cdot \left(1-x\right)$ (maximum $T_{\mathrm{up}}\cdot M_{p}$); research institution reward: $T_{\mathrm{rh}}\cdot M_{h}\cdot y$ (maximum $T_{\mathrm{rh}}\cdot M_{h}$); research institution penalty: $T_{\mathrm{rl}}\cdot M_{p}\cdot \left(1-y\right)$ (maximum $T_{\mathrm{rl}}\cdot M_{p}$).

When users upload honestly and research institutions store data properly, user revenue equals $R\cdot V\left(T\right)-C_{h}+T_{\mathrm{uh}}\cdot M_{h}\cdot x$, while research institution revenue equals $E_{h}-R\cdot V\left(T\right)+T_{\mathrm{rh}}\cdot M_{h}\cdot y$. If research institutions leak data (detection probability *P*), user revenue becomes $R\cdot V\left(T\right)-C_{h}-D+T_{\mathrm{uh}}\cdot M_{h}\cdot x$, with research institution revenue being $E_{h}-R\cdot V\left(T\right)+E_{l}-T_{\mathrm{rl}}\cdot M_{p}\cdot \left(1-y\right)\cdot P$.

For malicious users (detection probability *Z*), their expected revenue is $R\cdot \left(1-Z\right)\cdot V\left(T\right)-C_{p}-T_{\mathrm{up}}\cdot M_{p}\cdot \left(1-x\right)$. Research institutions obtain $-R\cdot \left(1-Z\right)\cdot V\left(T\right)+T_{\mathrm{rh}}\cdot M_{h}\cdot y$ when storing data honestly, or $-R\cdot \left(1-Z\right)\cdot V\left(T\right)-T_{\mathrm{rl}}\cdot M_{p}\cdot \left(1-y\right)\cdot P$ when leaking maliciously.

False EMRs provide no research value ($E_{h}=0$) but still trigger leakage penalties. The platform’s feedback mechanism ensures all malicious uploads eventually impact research institutions’ reputations, maintaining system integrity through consistent penalties regardless of data authenticity. This enforces behavioral compliance while preserving data credibility.

Based on the aforementioned analysis and following the approach by Sun [28] and Liu [29] for constructing the payoff matrix, we have established the payoff matrix for the model, as shown in Table 3.

### 5.2. Benefit Expectations of Users and Research Institutions

According to the constructed game matrix, we can calculate the expected returns of users and research institutions, respectively, when they adopt honest or malicious behaviors. Firstly, the formula for the expected gain of honest users is as follows:

(2) $$N_{\mathrm{honest}\;\mathrm{user}}=R\cdot V-D-C_{h}+D\cdot y+M_{h}\cdot T_{uh}\cdot x$$

The formula for calculating the expected revenue of a malicious user is as follows:

(3) $$\begin{array}{c} N_{\mathrm{Malicious}\;\mathrm{user}}=M_{p}\cdot T_{up}\cdot \left(x-1\right)-C_{p}-R\cdot V\cdot \left(Z-1\right) \end{array}$$

The average expected return for users is calculated as follows:

(4) $$\begin{array}{c} {\hat{N}}_{\mathrm{user}}=xN_{\mathrm{honest}\;\mathrm{user}}+\left(1-x\right)N_{\mathrm{malicious}\;\mathrm{user}}\\ =x\left(R\cdot V-D-C_{h}+D\cdot y+M_{h}\cdot T_{uh}\cdot x\right)\\ +\left(x-1\right)\left(C_{p}+M_{p}\cdot T_{up}-R\cdot V-M_{p}\cdot T_{up}\cdot x\right.\\ \left.+R\cdot V\cdot Z\right) \end{array}$$

The expected return for honest research institutions is as follows:

(5) $$\begin{array}{c} N_{\mathrm{honest}\;\mathrm{research}}=x\left(E_{h}-R\cdot V+M_{h}\cdot T_{rh}\cdot y\right)\\ -\left(M_{h}\cdot T_{rh}\cdot y+R\cdot V\cdot \left(Z-1\right)\right)\left(x-1\right) \end{array}$$

The expected return of the malignant research organization is as follows:

(6) $$\begin{array}{c} N_{\mathrm{malicious}\;\mathrm{research}}=\\ x\left(E_{h}+E_{l}-R\cdot V+M_{p}\cdot T_{rl}\cdot P\cdot \left(y-1\right)\right)\\ -\left(R\cdot V\cdot \left(Z-1\right)+M_{p}\cdot T_{rl}\cdot P\cdot \left(y-1\right)\right)\left(x-1\right) \end{array}$$

From this, the expected budgetary benefit of the evaluation of the research organization can be calculated as follows:

(7) $$\begin{array}{c} {\hat{N}}_{\mathrm{research}}=yN_{\mathrm{honest}\;\mathrm{research}}+\left(1-y\right)N_{\mathrm{malicious}\;\mathrm{research}}\\ =-y\left((M_{h}\cdot T_{rh}\cdot y+R\cdot V\cdot \left(Z-1\right))\left(x-1\right)\right.\\ \left.-x(E_{h}-R\cdot V+M_{h}\cdot T_{rh}\cdot y)\right)\\ -\left(x(E_{h}+E_{l}-R\cdot V+M_{p}\cdot T_{rl}\cdot P\cdot \left(y-1\right))\right.\\ \left.-(R\cdot V\cdot \left(Z-1\right)+M_{p}\cdot T_{rl}\cdot P\cdot \left(y-1\right))\left(x-1\right)\right)\\ \left.(y-1)\right) \end{array}$$

### 5.3. ESS Analyses by Users and Research Institutions

Within the framework of evolutionary game theory, *replicator dynamics* [37] (RD) establishes a foundational framework for analyzing strategy frequency dynamics in populations. Formally, it characterizes the temporal evolution of these frequencies through a differential equation:

$$\frac{dx}{dt}=x\left(N_{i}-\hat{N}\;\right)$$

In this equation, $x_{i}$ denotes the proportion of strategy *i* adopters in the population, $N_{i}$ corresponds to the strategy-specific payoff, and $\hat{N}$ represents the population average payoff.

The resulting dynamic equations for the user can be derived by bringing Equations (2)–(4) into the dynamic replication equation:

(8) $$\begin{array}{cc} F(x) & =\frac{dx}{dt}=x\left(N_{\mathrm{honest}\;\mathrm{user}}-{\hat{N}}_{\mathrm{user}}\right)\\ \; & =-x\left(x-1\right)\left(C_{p}-C_{h}-D+M_{p}\cdot T_{up}\right.\\ \; & \;\left.+D\cdot y+M_{h}\cdot T_{uh}\cdot x\right.\\ \; & \;\left.-M_{p}\cdot T_{up}\cdot x+R\cdot V\cdot Z\right) \end{array}$$

Similarly, bringing Equations (5)–(7) into the dynamic replication equation yields the dynamic equation for the research organization:

(9) $$\begin{array}{cc} F(y) & =\frac{dy}{dt}=y\left(N_{\mathrm{honest}\;\mathrm{research}}-{\hat{N}}_{\mathrm{research}}\right)\\ \; & =y\left(y-1\right)\left(E_{l}\cdot x-M_{p}\cdot T_{rl}\cdot P\right.\\ \; & \;\left.-M_{h}\cdot T_{rh}\cdot y+M_{p}\cdot T_{rl}\cdot P\cdot y\right) \end{array}$$

The dynamical system yields five equilibrium solutions from F(x)=F(y)=0:

$$E_{1}\left(0,0\right),\;E_{2}\left(0,1\right),\;E_{3}\left(1,0\right),\;E_{4}\left(1,1\right),\;\mathrm{and}\;E_{5}\left(x^{*},y^{*}\right),$$

where the interior equilibrium coordinates $x^{*}$ and $y^{*}$, defined by Equations (10) and (11), respectively, satisfy the biological feasibility condition $0\leq x^{*},y^{*}\leq 1$.

(10) $$\begin{array}{cc} x^{*} & =\frac{-(C_{p}-C_{h}-D+M_{p}\cdot T_{up}+D\cdot y+R\cdot V\cdot Z)}{M_{h}\cdot T_{uh}-M_{p}\cdot T_{up}} \end{array}$$

(11) $$\begin{array}{cc} y^{*} & =\frac{\left(E_{l}\cdot x-M_{p}\cdot T_{rl}\cdot P\right)}{\left(M_{h}\cdot T_{rh}-M_{p}\cdot T_{rl}\cdot P\right)}\\ \; & =1+\frac{E_{l}\cdot x-M_{h}\cdot T_{rh}}{M_{h}\cdot T_{rh}-M_{p}\cdot T_{rl}\cdot P} \end{array}$$

While the system exhibits five equilibrium points, only a subset of these satisfy the criteria for an evolutionarily stable strategy (ESS). To rigorously evaluate stability, we employ Jacobian matrix analysis [22]—a methodological cornerstone in evolutionary stability assessment. The eigenvalues of the Jacobian matrix at each equilibrium determine its stability classification under replicator dynamics.

The trace and determinant of the Jacobian matrix are key indicators for determining whether the RD system reaches an ESS:

- If det(J)>0 and tr(J)<0, the equilibrium satisfies the ESS condition;
- If det(J)<0, the equilibrium behaves as a saddle point;
- If det(J)>0 and tr(J)>0, the equilibrium is unstable.

We compute the partial derivatives of F(x) and F(y) with respect to *x* and *y*, respectively, obtaining the Jacobian matrix in (12):

(12) $$J=\left[\begin{array}{cc} C_{p}-C_{h}-D+M_{p}\cdot T_{up}+2\cdot C_{h}\cdot x- & \\ 2\cdot C_{p}\cdot x+2\cdot D\cdot x+D\cdot y & \\ +2\cdot M_{h}\cdot T_{uh}\cdot x-4\cdot M_{p}\cdot T_{up}\cdot x & -D\cdot x\cdot (x-1)\\ +R\cdot V\cdot Z-2\cdot D\cdot x\cdot y-3\cdot M_{h}\cdot T_{uh}\cdot x^{2} & \\ +3\cdot M_{p}\cdot T_{up}\cdot x^{2}-2\cdot R\cdot V\cdot x\cdot Z & \\ \; & M_{p}\cdot T_{rl}\cdot P-E_{l}\cdot x+2\cdot M_{h}\cdot T_{rh}\cdot y\\ E_{l}\cdot y\cdot \left(y-1\right) & +2\cdot E_{l}\cdot x\cdot y-3\cdot M_{h}\cdot T_{rh}\cdot y^{2}\\ \; & +3\cdot M_{p}\cdot T_{rl}\cdot P\cdot y^{2}-4\cdot M_{p}\cdot T_{rl}\cdot P\cdot y \end{array}\right]$$

According to Taylor and Jonker [38], the equilibrium $\left(x^{*},y^{*}\right)$ is a neutrally stable point of the system, exhibiting closed orbital trajectories in strategy selection for both users and research institutions. These trajectories remain in proximity to $\left(x^{*},y^{*}\right)$ without asymptotic convergence. Consequently, our stability analysis focuses on the corner equilibria $E_{1}\left(0,0\right)$, $E_{2}\left(0,1\right)$, $E_{3}\left(1,0\right)$, and $E_{4}\left(1,1\right)$. Substituting these equilibria into the Jacobian matrix yields four distinct trace-determinant pairs, as summarized in Table 4:

**Proposition** **1.** *$E_{1}\left(0,0\right)$ is a stable point if the following condition is satisfied:*

(13) $$\left\{\begin{array}{c} C_{p}-C_{h}-D+M_{p}\cdot T_{up}+R\cdot V\cdot Z<0\\ M_{p}\cdot T_{rl}\cdot P<0 \end{array}\right.$$

**Proof.**  When $C_{p}-C_{h}-D+M_{p}\cdot T_{up}+R\cdot V\cdot Z<0$ and $M_{p}\cdot T_{rl}\cdot P<0$, then det(J) has a positive sign at $E_{1}\left(0,0\right)$ and tr(J) has a negative sign at $E_{1}\left(0,0\right)$. Therefore, $E_{1}\left(0,0\right)$ satisfies the conditions of ESS, verifying the statement in Proposition 1. □

In scenarios where an anomalous incentive mechanism rewards research institutions for data leakage, such a mechanism would fundamentally undermine data protection principles and create systemic incentives for institutional data breaches. Furthermore, when a user’s net benefit calculation yields a negative value, i.e., $C_{p}-C_{h}-D+M_{p}\cdot T_{up}+R\cdot V\cdot Z<0$, this indicates an economic preference for uploading falsified data over genuine data due to unfavorable cost–benefit dynamics.

**Proposition** **2.** *$E_{2}\left(1,0\right)$ is a stable point if the following condition is satisfied:*

(14) $$\left\{\begin{array}{c} C_{h}-C_{p}+D-M_{h}\cdot T_{uh}-R\cdot V\cdot Z<0\\ M_{p}\cdot T_{rl}\cdot P-E_{l}<0 \end{array}\right.$$

**Proof.**  When $C_{p}-C_{h}-D+M_{p}\cdot T_{up}+R\cdot V\cdot Z<0$ and $M_{p}\cdot T_{rl}\cdot P-E_{l}<0,$ then det(J) has a positive sign at $E_{2}\left(1,0\right)$, and tr(J) has a positive sign at $E_{2}\left(1,0\right)$. det(J) has a positive sign and tr(J) has a negative sign at $E_{2}\left(1,0\right)$. Therefore, $E_{2}\left(1,0\right)$ satisfies the conditions of ESS, verifying the statement in Proposition 2. □

Through cost–benefit analysis, research institutions exhibit data leakage tendencies when the anticipated gains from breaching user data surpass the penalties associated with such violations. Conversely, user compliance with honest data submission emerges when the condition

R>L+ΔC

holds true, where *R* denotes integrity rewards, *L* represents potential leakage losses, and ΔC signifies the cost differential between maintaining data authenticity ($C_{\mathrm{real}}$) and fabricating information ($C_{\mathrm{false}}$). This equilibrium analysis ultimately converges to (1,0) as a stable strategic equilibrium point.

**Proposition** **3.** *$E_{3}\left(1,1\right)$ is a stable point when the following condition is satisfied:*

(15) $$\left\{\begin{array}{cc} C_{h}-C_{p}-M_{h}\cdot T_{uh}-R\cdot V\cdot Z & <0,\\ E_{l}-M_{h}\cdot T_{rh} & <0. \end{array}\right.$$

**Proof.**  When $C_{h}-C_{p}-M_{h}\cdot T_{uh}-R\cdot V\cdot Z<0$ and $E_{l}-M_{h}\cdot T_{rh}<0$, then det(J) has a positive sign at $E_{3}\left(1,1\right)$, and tr(J) has a negative sign at $E_{3}\left(1,1\right)$. Therefore, $E_{3}\left(1,1\right)$ satisfies the conditions of ESS, verifying the statement in Proposition 3. □

When research institutions are faced with the decision of whether to disclose user data, adopting a non-disclosure strategy suggests that the rewards from maintaining secrecy outweigh the potential value gains from data disclosure. For users, if the cost difference between uploading real data and uploading false data is smaller than the benefits obtained from honest uploading, they will find it economically advantageous to submit authentic information. This incentive structure motivates users to prefer providing truthful data over fabricated or misleading content. When both conditions are satisfied simultaneously, the system will reach a steady state where all users consistently choose honest data submission while research institutions opt to withhold disclosure of such data.

**Proposition** **4.** *$E_{4}\left(0,1\right)$ is a stable point when the following condition is satisfied:*

(16) $$\left\{\begin{array}{cc} C_{p}-C_{h}+M_{p}\cdot T_{up}+R\cdot V\cdot Z & <0,\\ -M_{h}\cdot T_{rh} & <0. \end{array}\right.$$

**Proof.**  When $C_{p}-C_{h}+M_{p}\cdot T_{up}+R\cdot V\cdot Z<0$ and $-M_{h}\cdot T_{rh}<0$, then det(J) has a positive sign at $E_{4}\left(0,1\right)$ and tr(J) has a negative sign at $E_{4}\left(0,1\right)$. Therefore, $E_{4}\left(0,1\right)$ satisfies the conditions of ESS, verifying the statement in Proposition 4. □

When the research institution’s confidentiality preservation benefit exceeds zero, whereas the net cost advantage of honest data submission (calculated as honest upload cost minus malicious upload cost) remains negative relative to the combined value of leakage penalties and honest behavior incentives, rational users will exhibit a preference for malicious data submission. Under these conditions, users perceive the marginal benefit of honest participation as insufficient to offset the operational efficiency gains from data manipulation, even when accounting for potential confidentiality breach risks. This strategic interaction converges to a Nash equilibrium wherein (1) systematic malicious data submission becomes the dominant user strategy, and (2) research institutions rationally maintain confidentiality through non-disclosure protocols.

Based on the analysis provided, it can be concluded that E1(0,0), E2(1,0), E3(1,1), and $E4\left(0,1\right)$ can be used as stabilization points for the EMRs platform under certain conditions. However, based on the fact that the initial purpose of the EMRS platform is to promote both users and research institutions to choose honest strategies, we will focus only on this case of $E3\left(1,1\right)$, which means that the data of the system has to satisfy the conditions of Proposition 3.

## 6. Numerical Simulation

Numerical simulations play a crucial role in game theory research, providing us with insights and clear evolutionary trends. Our goal is to dissect how the initial conditions shape the final game outcome and to delve into the impact of the following key parameters on the evolutionary path of the game: the probability *Z* that the EMRs platform checks for malicious uploads by users, the probability *P* that the EMRs platform detects data leakage behavior by research institutes, the reward coefficients *V* that are dynamically adjusted based on the user’s reputation, and the dynamic equations of the reward coefficients $M_{h}$ and the penalty coefficient $M_{p}$.

**Figure 2:**
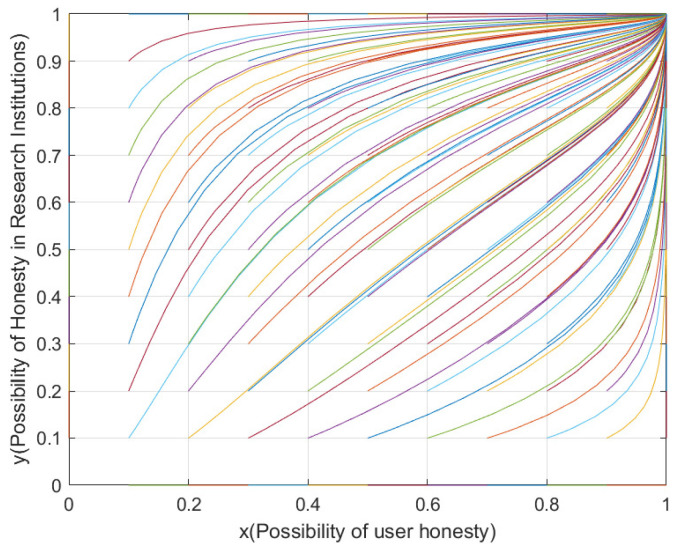
System evolutionary trajectories.

Based on the framework proposed by Sun [29], we integrate the parameter interaction analysis methodology and replicate other models as shown in Table 5. The evolutionary results are demonstrated in Figure 2.

### 6.1. Impact of P on the Evolution of Scientific Institutions

The user’s willingness to upload honestly is set to 0.5, and the initial confidentiality willingness of research institutions is set to three different levels from low to high, i.e., $y\in \left(0.1,0.4,0.7\right)$. When adjusting *P*, the impact on the evolutionary outcomes for research institutions with different initial willingness to keep secrets is explored.

Initial honesty intention [39], derived from psychological research on intrinsic behavioral tendencies, refers to the likelihood of users honestly sharing medical data during the initial phase in this study. This probability reflects their baseline psychological state and serves as the foundational input for modeling behavioral evolution. While subsequent actions may be dynamically influenced by factors such as incentive mechanisms and perceived privacy risks, the initial intention establishes the baseline trajectory for the evolutionary path.

**Figure 3:**
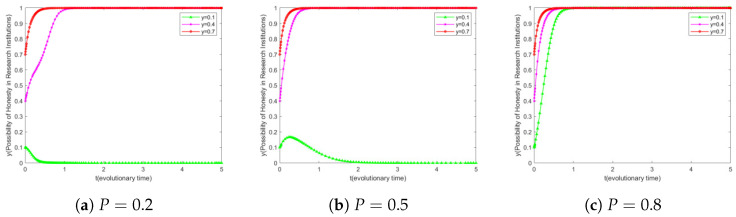
Simulation results of the evolution of scientific institutions.

As shown in Figure 3a, checking the probability P=0.2 of malicious disclosure by research institutions, we observe that research institutions with a low initial willingness to keep secrets (y=0.1) eventually evolve to fully disclose their data, whereas those with a high willingness to keep secrets (y=0.4 or y=0.7) are able to maintain and enhance their willingness to maintain secrecy and eventually reach a state of complete secrecy. This phenomenon reveals the relationship between the probability of detection and the initial willingness to keep secrets: At a low probability of detection, institutions with a low initial willingness to keep secrets may choose to leak because the potential benefits of leaking the data outweigh the penalties of being detected, while those with a high initial willingness to keep secrets are able to resist the temptation of leaking and maintain their secretive behavior.

Comparing Figure 3a,b, it can be seen that when P=0.5, y=0.2. Research institutions have a different evolutionary path compared to those at P=0.2. In the initial evolutionary stage, their willingness to evolve tends to be honest, but when the evolutionary time is about 0.2, their willingness to act reverses and tends to be malicious. This trend continues to evolve over time, and eventually, the research institution’s willingness may be completely inclined towards malicious behavior. This suggests that *P* has an effect on the behavior of research institutions to some extent, but if *P* is not high enough, the incentive it provides may not be sufficient to support the full evolution of research institutions towards honest behavior.

Further analyzing Figure 3b,c, after *P* rises from 0.5 to 0.8, we can see that the research institutions with an initial willingness to keep secrets of 0.1 undergo a significant transition from gradually decreasing to 0 in Figure 3b to gradually evolving to converging to 1 in Figure 3c. This suggests that, with the increase in the probability of detection, the platform’s detection and penalty mechanism has a significant guiding and constraining effect on the research institutions initially less willing to keep secrets with a significant guiding and constraining effect.

Further analysis shows that the detection probability (P) of monitoring unauthorized resale by research institutions is crucial to their motivation to keep research confidential. The key to boosting *P* is to strengthen the oversight of blockchain-based transactions. This not only improves monitoring efficiency but also significantly reinforces the confidentiality motivation of research institutions. Thus, it helps build a more robust research-data-management system.

### 6.2. Impact of *Z* on User Evolution

In this study, we set the research organization’s willingness to keep secrets as medium level, i.e., y=0.5. Based on this, we classify user honesty willingness into three levels, namely, low (x=0.1), medium (x=0.5), and high (x=0.8), in order to cover all stages of user honesty willingness. Further, this study adjusts the probability *Z* of the EMRs platform to detect repeated uploading behaviors of users to explore the results of user behaviors evolving under different initial willingness levels.

**Figure 4:**
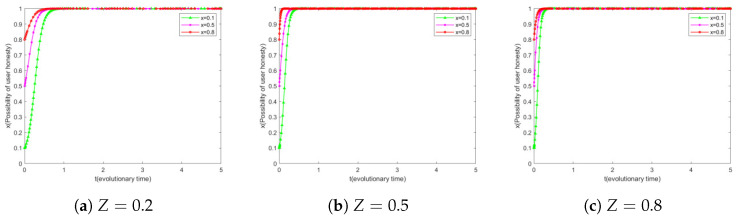
*Z* simulation results of different simultaneous user evolution.

Figure 4a reveals that users with different initial honesty willingness levels eventually evolve to full honesty (i.e., honesty willingness of 1) at detection probability Z=0.2, with users with an initial honesty willingness of x=0.1 taking significantly longer to evolve than users with other levels. By comparing Figure 4a–c, we can observe that when the detection probability *Z* is increased from 0.2 to 0.5, the evolutionary speed of users with initial honesty willingness of x=0.1 is significantly increased. However, when *Z* is further increased to 0.8, this speedup is not as significant as before. This suggests that the guiding and incentivizing effect on users’ honesty willingness increases gradually as the probability of detection increases, but for users with a low initial honesty willingness, the marginal benefit of this incentive effect may diminish when *Z* reaches a high level.

By comparing Figure 3a and Figure 4a, we can observe that with a low probability of successful detection *P* and *Z*, research institutions with a low initial willingness to be secretive may give up secrecy completely, i.e., y=0. In contrast, users with a low initial willingness to be honest may gradually evolve to full honesty. This differential response reveals the different sensitivities of research institutions and users to the detection mechanism.

For research institutions, their secrecy behavior is directly related to the detection success rate *P*. A lower detection success rate reduces the risk that a research institution’s leaking behavior will be identified, which may facilitate the propensity for data leakage. In this scenario, the leaking behavior of research institutions may go undetected for a long time, which underlines the importance of detection mechanisms in curbing data leakage.

For users, honest information rewards are associated with a *Z* probability of successful checking. The user’s motivation to upload honest information stems partly from the rewards offered by the system and partly from the system’s reputation rewards and penalties. Since the user’s reputation reward and punishment is based on the feedback from the research institution on the authenticity of the data to the EMRs platform, the user will be punished with a corresponding reputation penalty if he/she chooses to act maliciously. The existence of this mechanism provides users with a continuous incentive to remain honest, even when the success rate of the test is low.

In summary, the different responses of research institutions and users to detection mechanisms suggest that the characteristics and needs of different subjects need to be taken into account when designing incentive and punishment mechanisms. The confidential behavior of research institutions is more likely to be directly affected by detection success, while the honest behavior of users is driven by incentive mechanisms and constrained by reputation penalties.

### 6.3. Impact of *V* on User Evolution

The research institution’s willingness to keep secrets is set to 0.5, and the user’s willingness to be honest is set to three levels from low to high, i.e., $x\in \left(0.1,0.5,0.8\right)$. On this basis, we adjusted the reward coefficient V to explore the differences in user evolutionary outcomes under different levels of initial honesty willingness.

**Figure 5:**
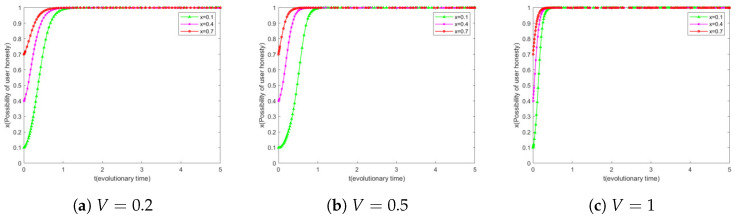
Simulation results of *V* different simultaneous user evolutions.

Figure 5a shows that users with a higher initial honesty willingness exhibit faster and more stable evolutionary rates for the same reward coefficients. For users with a lower initial honesty willingness, e.g., x=0.1, the rate of the increase in their honesty willingness is initially slower, but the rate of evolution accelerates significantly when evolving to approximately x=0.4. Figure 5b,c further reveal that users with an initial honest willingness of x=0.1 have a significantly higher rate of evolution in honest willingness when the reward coefficient V increases from 0.2 to 0.5. For users with an initial honesty willingness of x=0.5 and x=0.8, the rate of evolution increases but the enhancement is not as significant as for users with a lower initial honesty willingness. When the reward coefficient V is further increased from 0.5 to 1, the enhancement of the evolutionary rate is not significant for all three different levels of users.

These findings suggest that adopting a higher *V* in the initial stage of the system can effectively promote the evolution of users’ honesty willingness. As the user’s reputation level increases, the reward coefficient should be adjusted in due course to achieve the goal of motivating the user to shift to honest behavior at minimal cost. This provides strategic guidance for system designers to set the reward mechanism reasonably to optimize the incentive effect at different stages of user behavior evolution.

### 6.4. Effect of $M_{h}$ and $M_{p}$ on System Evolution

The user’s willingness to be honest x and the research institution’s willingness to keep secrets *y* are set to 0.2, and the effects of the changes in the values of $M_{h}$ and $M_{p}$ on the evolution of the user and the research institution are analyzed respectively, to explore the different motivational effectiveness of different reward and punishment coefficients for the user and the research institution.

**Figure 6:**
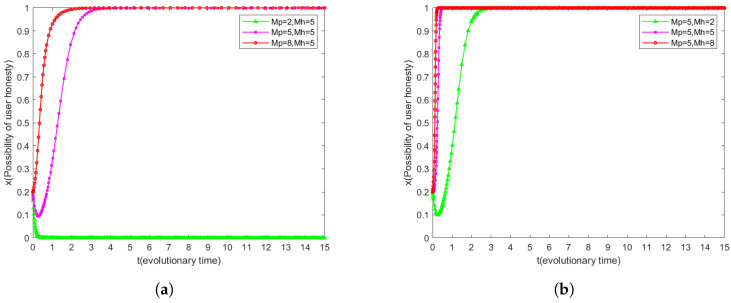
Dynamic strategies of users under varying penalty and reward coefficients. (**a**) Dynamic user strategies when penalty coefficients vary. (**b**) Users’ dynamic strategies when reward coefficients change.

As shown in Figure 6a, users’ willingness to be honest increases with the increase in the reward coefficient, but if the reward coefficient is too low, users’ willingness to be honest may gradually decrease to 0. Figure 6b further confirms the positive effect of the increase of the reward coefficient on users’ willingness to be honest. Comparing Figure 6a and Figure 6b, we find that lower punishment coefficients may lead to users’ tendency to be dishonest. Nevertheless, even with a lower reward coefficient, users tend to be honest over time after an initial decline in their willingness to be honest. This suggests that an increase in the penalty coefficient is more effective than the reward coefficient in preventing users from being dishonest.

However, under a particular combination of reward and punishment coefficients, e.g., a reward coefficient of $M_{h}$ = 8 and a punishment coefficient of $M_{p}$ = 5, higher reward coefficients promote users’ evolution towards honesty faster compared to a reward coefficient of $M_{h}$ = 5 and a punishment coefficient of $M_{p}$ = 8. This finding suggests that higher reward coefficients are more sensitive to users’ honest evolution under the same conditions.

Therefore, while ensuring that users tend to be honest, priority should be given to increasing the reward coefficient in order to promote the honest evolution of user behavior. This strategy not only incentivizes users to remain honest, but also achieves this goal at a manageable system cost.

**Figure 7:**
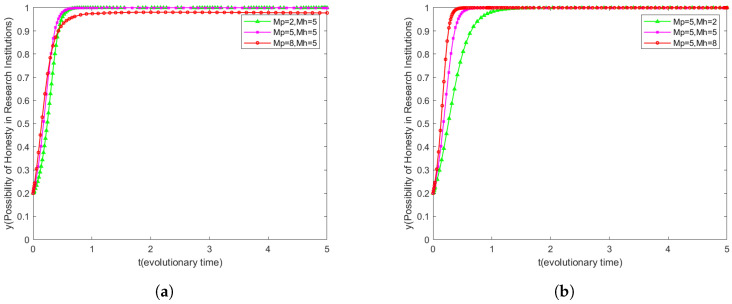
Dynamic strategies of scientific institutions under varying conditions. (**a**) Dynamic strategies of scientific institutions when the penalty coefficient changes. (**b**) Dynamic strategies of scientific institutions when the incentive factor changes.

The effect of the penalty coefficient $M_{p}$ on the leakage willingness of research institutions is revealed in Figure 7a. Unlike the case of users in Figure 6a, the willingness to leak of research institutions does not increase significantly when the penalty coefficient $M_{p}$ is low. This difference stems from the fact that research institutions assess potential secondary gains differently from users.

Further comparing Figure 7a,b, we can observe that the incentive effect of the reward coefficient $M_{h}$ is not as significant as that of the penalty coefficient $M_{p}$ for research institutions. This finding points to the fact that research institutions are highly attracted to the secondary benefits gained from leaking user data, and, therefore, higher leakage penalties are more effective in motivating research institutions to keep user data confidential.

In summary, penalty coefficients play a more critical role in the incentives for confidentiality behavior in research institutions. This emphasizes that when designing data protection policies for research institutions, more consideration should be given to how the willingness to maintain confidentiality can be enhanced by increasing the penalty cost of leakage, rather than relying solely on incentive mechanisms.

### 6.5. Discussion

We analyze the impact of different conditions on the honest behavior of users and research institutions by constructing a two-sided evolutionary game model for users and research institutions. A key result of the analysis is that higher *P* has a significant impact on the evolutionary outcomes for research institutions. Specifically, when *P* is higher, research institutions show a stronger tendency to evolve honestly. This finding highlights the importance and necessity of higher *P* in EMRs platforms during the initial setup phase in deterring research institutions from leaking data.

In this context, our study also analyzes the impact of the probability *Z*, the reward coefficient *V*, the dynamic reward coefficient $M_{h}$, and the dynamic penalty coefficient $M_{p}$ on the evolutionary outcomes of EMRs platforms, respectively. For example, a higher *Z* makes users more inclined to be honest, but the effect on users is not significant after it is higher than 0.5. Interestingly, lower penalty coefficients do not make research institutions inclined to be malicious, but they do make users inclined to be malicious. Lower reward coefficients, on the other hand, only affect the rate of evolution to honesty for both users and research institutions, and do not make them inclined to malice. These findings contribute to the design of effective incentives in EMRs platforms, emphasizing the need to carefully consider these parameters.

## Figures and Tables

**Table 1:** Comparison of existing blockchain EMRs incentive mechanisms.

Research	Target of Incentive	Incentive Measure	Incentive Differentiation	Dynamic Adaptability
[26]	Users	Token Reward	×	×
[19]	Users + Research institution	Dynamic Reputation	✓	×
[20]	Users + Research institution	Dynamic Reputation	✓	×
[21]	Research institution	Dynamic Reputation + Reward	✓	×
This Paper	Users + Research institution	Dynamic Reputation + Reward	✓	✓

**Table 2:** Parameters in the model.

Variate	Description
*Z*	Probability of EMRs platform checking out a user’s malicious uploads
*P*	Probability of EMRs platform checking the probability of malicious leaks from research organizations
$C_{p}$	The cost of malicious data uploads by users
$C_{h}$	The cost to the user of honestly uploading data
$E_{l}$	Benefits of malicious disclosure of real user data by research organizations
$E_{h}$	Benefits to research organizations from users uploading honest data
*R*	Maximum incentives paid to users by research organizations
*D*	Losses to users caused by the leakage of real user data by research organizations
*T*	Participant’s current credit score
$T_{rh}$	Credibility of honest secrecy system rewards for research organizations
$T_{rl}$	Reputation for systematic penalties in the event of malicious disclosure by research organizations
$T_{uh}$	Reputation rewarded by the system for honest uploads by users
$T_{up}$	Reputation for system penalties in case of malicious user leakage
$T_{starting}$	Credibility of initial system settings
*V*	The coefficient by which the platform rewards users based on their reputation in the case of dynamic rewards and penalties
$M_{h}$	System dynamic incentive factor
$M_{p}$	System dynamic penalty factor

**Table 3:** The payoff matrix of the participating roles.

		User	
		Honest (x)	Malicious (1−x)
**Research organization**	Honest (*y*)	$E_{h}-R*V\left(T\right)+T_{rh}*M_{h}*y$, $R*V\left(T\right)-C_{h}+T_{uh}*M_{h}*x$	$-R*\left(1-Z\right)*V\left(T\right)+T_{rh}*M_{h}*y$, $R*\left(1-Z\right)*V\left(T\right)-C_{p}-T_{up}*M_{p}*\left(1-x\right)$
	Malicious (1−y)	$E_{h}-R*V\left(T\right)+E_{l}-T_{rl}*M_{p}*\left(1-y\right)*P$, $R*V\left(T\right)-C_{h}-D+T_{uh}*M_{h}*x$	$-R*\left(1-Z\right)*V\left(T\right)-T_{rl}*M_{p}*\left(1-y\right)*P$, $R*\left(1-Z\right)*V\left(T\right)-C_{p}-T_{up}*M_{p}*\left(1-x\right)$

**Table 4:** The system’s tr(J) and det(J).

Equilibrium Point	detJ	trJ
$E_{1}\left(0,0\right)$	$\left(C_{p}-C_{h}-D+M_{p}T_{up}+RVZ\right)\left(M_{p}T_{rl}P\right)$	$\left(C_{p}-C_{h}-D+M_{p}T_{up}+RVZ\right)$
$E_{2}\left(1,0\right)$	$\left(C_{h}-C_{p}+D-M_{h}T_{uh}-RVZ\right)\left(M_{p}T_{rl}P-E_{l}\right)$	$\left(C_{h}-C_{p}+D-M_{h}T_{uh}-RVZ\right)$
$E_{3}\left(1,1\right)$	$\left(C_{h}-C_{p}-M_{h}T_{uh}-RVZ\right)\left(E_{l}-M_{h}T_{rh}\right)$	$\left(C_{h}-C_{p}-M_{h}T_{uh}-RVZ\right)$
$E_{4}\left(0,1\right)$	$\left(C_{p}-C_{h}+M_{p}T_{up}+RVZ\right)\left(-M_{h}T_{rh}\right)$	$\left(C_{p}-C_{h}+M_{p}T_{up}+RVZ\right)$

**Table 5:** The remaining parameter values of the experimental assumptions.

$C_{p}$	$C_{h}$	$E_{l}$	$E_{h}$	*R*	*D*	$T_{rh}$	$T_{rl}$	$T_{up}$	$T_{uh}$
4	24	15	40	30	10	3	3	3	3

## Data Availability

Data are contained within the article.
